# Increased dietary α-linolenic acid has sex-specific effects upon eicosapentaenoic acid status in humans: re-examination of data from a randomised, placebo-controlled, parallel study

**DOI:** 10.1186/1475-2891-13-113

**Published:** 2014-12-11

**Authors:** Caroline E Childs, Samantha Kew, Yvonne E Finnegan, Anne M Minihane, Elizabeth C Leigh-Firbank, Christine M Williams, Philip C Calder

**Affiliations:** Human Development and Health Academic Unit, Faculty of Medicine, University of Southampton, MP887 Southampton General Hospital, Tremona Road, Southampton, SO16 6YD UK; Hugh Sinclair Unit of Human Nutrition, Department of Food and Nutritional Sciences, The University of Reading, Reading, RG6 6AP UK; Department of Nutrition, Norwich Medical School, University of East Anglia, Norwich, NR4 7TJ UK; NIHR Southampton Biomedical Research Centre, University Hospital Southampton NHS Foundation Trust and University of Southampton, Southampton, SO16 6YD UK; Department of Biological Sciences, Faculty of Science, King, Abdulaziz University, Jeddah, Saudi Arabia

**Keywords:** α-linolenic acid, Sex, Eicosapentaenoic acid

## Abstract

**Background:**

There is a metabolic pathway by which mammals can convert the omega-3 (n-3) essential fatty acid α-linolenic acid (ALA) into longer-chain n-3 polyunsaturated fatty acids (LC n-3 PUFA) including eicosapentaenoic acid (EPA) and docosahexaenoic acid (DHA). As far as we know there are currently no studies that have specifically examined sex differences in the LC n-3 PUFA response to increased dietary ALA intake in humans, although acute studies with isotope-labelled ALA identified that women have a significantly greater capacity to synthesise EPA and DHA from ALA compared to men.

**Findings:**

Available data from a placebo-controlled, randomised study were re-examined to identify whether there are sex differences in the LC n-3 PUFA response to increased dietary ALA intake in humans. There was a significant difference between sexes in the response to increased dietary ALA, with women having a significantly greater increase in the EPA content of plasma phospholipids (mean +2.0% of total fatty acids) after six months of an ALA-rich diet compared to men (mean +0.7%, P = 0.039). Age and BMI were identified as predictors of response to dietary ALA among women.

**Conclusions:**

Women show a greater increase in circulating EPA than men during increased dietary ALA consumption. Further understanding of individual variation in the response to dietary ALA could inform nutrition advice, with recommendations being specifically tailored according to habitual diet, sex, age and BMI.

## Findings

### Background

There is a metabolic pathway by which mammals can convert the omega-3 (n-3) essential fatty acid α-linolenic acid (ALA; 18:3n-3) into longer-chain (LC) more unsaturated n-3 polyunsaturated fatty acids (PUFA) such as eicosapentaenoic acid (EPA; 20:5n-3), docosapentaenoic acid (DPA; 22:5n-3) and docosahexaenoic acid (DHA; 22:6n-3) via a series of desaturase and elongase catalysed reactions [1]. Acute studies with stable isotope-labelled ALA have identified that females have a significantly greater capacity than males to synthesise EPA and DHA from ALA, with estimated net conversion rates of ALA to EPA of 21% vs. 8% and of ALA to DHA of 9% vs. 0% [2, 3]. Correspondingly, females have been found to have significantly higher concentrations of DHA in plasma lipids and erythrocytes compared to males, regardless of their dietary intake of n-3 fatty acids [4]. Sex differences in EPA and DPA content have also been observed, with females having higher erythrocyte phospholipid EPA, lower adipose tissue EPA and lower plasma DPA content [4]. Studies of women using the contraceptive pill [5, 6] or hormone-replacement therapy [7, 8] and of trans-sexual subjects [6] suggest that sex hormones play a role in mediating these sex differences in LC n-3 PUFA status.

As far as we know there are currently no studies that have specifically examined sex differences in the fatty acid response to a chronic increase in dietary ALA intake in humans, with available data from male or mixed sex cohorts indicating modest but significant increases in circulating EPA and but not DHA, following increased ALA intake [9–11]. If there are significant sex differences in the response to increased ALA intake in humans, this must be considered during trials and has implications for dietary advice and recommendations. Here we re-examine data from a human dietary study [12] to test our hypothesis that sex can significantly influence the increment in LC n-3 PUFA status following ALA supplementation.

### Methods

Data selected for secondary analysis were plasma phospholipid fatty acids from a study of healthy male (M; n = 87) and female (F; n = 63) participants aged 25–72 yr who had replaced their normal margarine or butter with specially formulated margarines for a period of 6 months [12]. The original study involved randomisation of participants to one of five groups, including a control group who consumed a standard high linoleic acid, low ALA margarine, two groups who consumed margarines enriched with ALA, and two groups who consumed margarines enriched with EPA + DHA [12]. Here data from the control and the “high-ALA” groups are used. The high-ALA margarine provided contained 41 g ALA/100 g compared with 1 g ALA/100 g in the control margarine. The study was approved by the University of Reading Ethics and Research Committee and the West Berkshire Health Authority Ethics Committee, and each participant gave written informed consent before participating. Participants in the control group (n = 30; 18 M, 12 F) consumed an average of 1.5 g ALA/day (from background diet and the control margarine), and those receiving the high-ALA margarine (n = 29; 17 M, 12 F) consumed an average of 9.5 g ALA/day (from background diet and the high-ALA margarine) over the 6 month period [12]. Available data for which paired baseline and 6 month values were available were included in this analysis (control group n = 21; high-ALA group n = 23). The data set supporting the results of this article is available in the University of Southampton Institutional Research Repository [ePrints Soton: http://eprints.soton.ac.uk/id/eprint/370208].

Lipid was extracted from plasma with chloroform:methanol (2:1, by vol) and phospholipids were isolated by thin-layer chromatography by using a mixture of hexane:diethyl ether:acetic acid (90:30:1, by vol) as the elution phase. Fatty acid methyl esters were prepared by incubation with 14% boron trifluoride at 80°C for 60 min. Fatty acid methyl esters were separated in a gas chromatograph and identified by comparison with standards.

Data were analysed using IBS SPSS Statistics 20 (IBM, Portsmouth, UK). Data are expressed as change from baseline following the six month dietary period and were assessed for the effect of diet, sex and diet*sex interactions by two-way ANOVA. Where significant effects were found, differences between dietary groups and the sexes were explored by independent T tests. Linear regression was conducted to investigate the influence of diet, age, weight and BMI upon the change in EPA content.

### Results

Males weighed significantly more than females within both dietary groups, resulting in a significantly higher intake of ALA (expressed as g per kg body weight) among females in both dietary groups (Table 1). No sex differences were identified for any n-3 PUFA at baseline (Table 1). Participants randomised to the high-ALA diet had a significantly lower baseline DHA status than those in the control group (Table 1).

**Table 1:** **Characteristics of participants in the study according to diet**

	1.5 g/d ALA		9.5 g/d ALA		P value		
	Male	Female	Male	Female	Sex	Diet	Sex*diet
	(n = 11)	(n = 10)	(n = 13)	(n = 10)			
Age (y)	52.9 ± 13.7	53.0 ± 11.2	50.5 ± 12.7	53.5 ± 12.0	0.68	0.80	0.70
Weight (kg)	82.3 ± 8.8	62.9 ± 7.4*	84.7 ± 14.3	69.9 ± 9.2*	<0.001	0.15	0.47
BMI (kg/m^2^)	26.4 ± 2.7	24.2 ± 3.1	27.3 ± 3.9	25.6 ± 2.6	0.053	0.24	0.82
ALA dose per kg body weight (mg/d)	18.4 ± 1.9	24.2 ± 2.6*	115.5 ± 22.0^†^	137.8 ± 16.8^†^*	0.003	<0.001	0.067
Baseline plasma phospholipid n-3 fatty acid status (% total fatty acids)							
ALA (18:3n-3)	0.3 ± 0.1	0.4 ± 0.1	0.3 ± 0.1	0.3 ± 0.1	0.63	0.20	0.75
EPA (20:5n-3)	0.9 ± 0.8	1.1 ± 0.9	0.8 ± 0.6	0.8 ± 0.5	0.55	0.48	0.73
DPA (22:5n-3)	2.1 ± 1.5	1.6 ± 0.7	1.9 ± 0.7	1.8 ± 0.9	0.34	0.98	0.63
DHA (22:6n-3)	4.4 ± 1.3	4.2 ± 1.3	3.5 ± 1.0	3.2 ± 0.9	0.63	0.008	0.86

Data are mean ± SD.

*Significantly different from males in same dietary group (p < 0.05, independent mean t-test). ^†^Significant effect of diet within same sex (p < 0.05, independent mean t-test).

The diet provided significantly altered plasma phospholipid n-3 fatty acid content, with those receiving the high-ALA diet having significantly higher ALA and EPA contents **(**Table 2). Significant sex*diet interactions were observed for plasma phospholipid 16:0, 20:1n-9 and EPA content. Females receiving the high-ALA diet had a significantly greater increase in plasma phospholipid EPA than males consuming the same diet (Table 2). Women showed a mean 2.0% of total fatty acids increase in the EPA content of plasma phospholipids after the high-ALA diet compared with a mean 0.7% increase in men (P = 0.039). No significant sex*diet interaction was identified for any other n-3 PUFA, including DHA, or for any n-6 PUFA (Table 2). Inclusion of participant age, BMI or weight in the model as covariates did not alter the pattern of results observed for plasma phospholipid EPA content (data not presented).

**Table 2:** **Plasma phospholipid fatty acid composition after control or ALA-rich diet for 6 months**

	1.5 g/d ALA		9.5 g/d ALA				
	Male	Female	Male	Female		P value ^1^	
	(n = 11)	(n = 10)	(n = 13)	(n = 10)	Sex	Diet	Sex*diet
	*(% total fatty acids)*						
16:0	26.7 ± 1.5	27.5 ± 1.4	28.0 ± 2.2	26.8 ± 1.3	0.82	0.44	0.073
18:0	13.2 ± 1.2	13.4 ± 1.5	12.5 ± 3.8	13.6 ± 1.6	0.82	0.84	0.20
22:0	1.4 ± 0.9	1.0 ± 0.5	1.3 ± 1.0	1.4 ± 1.1	0.11	0.50	0.69
18:1n-9	10.8 ± 3.0	10.9 ± 2.3	11.4 ± 2.9	11.2 ± 1.9	0.78	0.53	0.64
20:1n-9	0.4 ± 0.3	0.2 ± 0.1*	0.4 ± 0.2	0.3 ± 0.2	0.18	0.39	0.041
18:2n-6	22.2 ± 1.8	22.3 ± 2.4	22.1 ± 2.5	21.6 ± 2.6	0.55	0.58	0.61
18:3n-6	0.2 ± 0.2	0.2 ± 0.2	0.1 ± 0.1	0.2 ± 0.1	0.60	0.14	0.60
20:3n-6	3.6 ± 1.0	4.2 ± 1.3	3.5 ± 1.4	3.0 ± 1.6	0.97	0.38	0.12
20:4n-6	10.3 ± 2.4	10.0 ± 2.0	9.1 ± 2.7	8.8 ± 1.3	0.74	0.26	0.32
18:3n-3	0.3 ± 0.1	0.3 ± 0.2	0.6 ± 0.3^†^	0.9 ± 0.3^†^	0.19	<0.001	0.11
20:5n-3	1.5 ± 1.5	1.2 ± 1.0	1.5 ± 1.3	2.8 ± 1.7^†^*	0.35	0.023	0.027
22:5n-3	2.0 ± 0.5	2.3 ± 0.7	2.5 ± 0.7	2.6 ± 0.7	0.11	0.22	0.39
22:6n-3	4.7 ± 1.2	3.9 ± 0.6	3.9 ± 1.3	3.7 ± 1.1	0.34	0.20	0.36

Data are mean ± SD.

^1^Statistics were conducted using change from baseline data. *Significantly different from males in same dietary group (p < 0.05, independent mean t-test). ^†^Significant effect of diet within same sex (p < 0.05, independent mean t-test).

Linear regression was conducted within each sex to determine the contribution of diet, age, weight and BMI to the change in plasma phospholipid EPA status. Among females, diet, age and BMI were significant predictors of the change in plasma phospholipid EPA content (Table 3). Age was inversely related to change in EPA status among females, while diet and BMI were significantly positively related with change in plasma phospholipid EPA status. None of these variables was a significant predictor of the change in plasma phospholipid EPA in men (Table 3). Data were examined for correlations between estimated ALA intake expressed as mg/d/kg bodyweight and change in EPA content in order to assess whether a higher relative dose was responsible for the sex differences observed. No significant correlations were observed (data not presented).

**Table 3:** **Linear regression analysis of change in plasma phospholipid EPA status** ^1^

	Males	P	Females	P
	(n = 24)		(n = 20)	
Model R	0.28	0.80	0.79	0.004
Standardised coefficients (Beta)				
BMI	−0.06	0.88	1.06	0.008
Diet (1.5 g/d ALA vs. 9.5 g/d ALA)	0.02	0.94	0.63	0.003
Age	−0.15	0.56	−0.47	0.029
Weight	−0.15	0.68	−0.70	0.055

^1^Data used are from participants in both the control group (1.5 g ALA/day) and the high ALA group (9.5 g ALA/day).

### Discussion and conclusions

These data demonstrate that there are sex differences in the response to dietary ALA in humans, with females having a significantly greater increase in EPA content of plasma phospholipids compared to males over the same period on the same diet. This confirms that the sex differences observed in the synthesis of EPA from ALA in acute studies using stable isotopes [2, 3] are also reflected in the response to chronic dietary regimes involving increased dietary supply of ALA. There was no sex specific enhancement in DPA or DHA content of plasma phospholipids after chronically increased ALA intake, nor any significant sex differences in DPA or DHA at baseline.

Data were not available on the menopausal status of women in the study, and insufficient data were available to perform a subset analysis of those participants under 45 years old (Females < 45 yr: 1.5g/d ALA n = 1, 9.5g/d ALA n = 2). However, an inverse relationship between the age of female participants and the change in EPA content after supplementation was observed, supportive of a role for female sex hormones in regulating the endogenous synthesis of LC n-3 PUFA. Larger studies will be required to fully investigate the role that menopausal status has upon the sex differences observed in this analysis. Data from this analysis indicates that a diet rich in ALA does not contribute to circulating DHA status, and the consequences of this should be considered in circumstances where there may be specific needs for DHA, such as during pregnancy.

The observation that BMI was positively correlated to change in plasma phospholipid EPA content may have two explanations. Firstly, increasing BMI is typically indicative of increasing body fatness and so of a proportional decrease in lean mass. This may result in less use of ALA for oxidation with increasing fatness, sparing a greater proportion of the ALA consumed to be used for conversion to LC n-3 PUFA. Secondly adipose tissue may have a role in the synthesis of LC PUFA, either as a primary source of endogenous synthesis, or indirectly via the higher circulating estradiol status associated with increasing adiposity. For these same reasons, relative adiposity may also contribute to sex differences in fatty acid status, as female fat mass is significantly higher than that of males with the same BMI. Thus, young women may have a greater capacity to respond to an increased intake of ALA than males, and may therefore have a lower need for preformed dietary EPA. Further studies incorporating additional measures indicative of body composition and adiposity, such as waist circumference will be required to further investigate these hypotheses.

Further understanding of the sex-linked variation in the response to dietary ALA could inform future dietary recommendations, with advice specifically tailored to match that individual’s sex. Studies which investigate other factors known to influence PUFA metabolism such as age, sex hormone status, adiposity and genotype may further identify those people who are most likely to benefit from ALA supplementation and inform personalised nutrition recommendations. The current recommended intake of EPA + DHA for adults in the UK is a minimum of 450 mg/day, yet it is estimated that over 70% of UK adults do not habitually consume any oily fish [13]. If a diet rich in ALA can contribute to EPA status in women, this could both inform dietary advice to women, especially those unable or unwilling to consume oily fish, and provide evidence for an alternative and sustainable dietary source of n-3 fatty acids.

## Abbreviations

ALA: α-linolenic acid
DHA: Docosahexaenoic acid
DPA: Docosapentaenoic acid
EPA: Eicosapentaenoic acid
F: Female
LC: Longer-chain
M: Male
PUFA: Polyunsaturated fatty acids.

## References

[1] Sprecher H (2000). Metabolism of highly unsaturated n-3 and n-6 fatty acids. Biochim Biophys Acta.

[2] Burdge GC, Jones AE, Wootton SA (2002). Eicosapentaenoic and docosapentaenoic acids are the principal products of alpha-linolenic acid metabolism in young men. Br J Nutr.

[3] Burdge GC, Wootton SA (2002). Conversion of alpha-linolenic acid to eicosapentaenoic, docosapentaenoic and docosahexaenoic acids in young women. Br J Nutr.

[4] Lohner S, Fekete K, Marosvolgyi T, Decsi T (2013). Gender differences in the long-chain polyunsaturated fatty acid status: systematic review of 51 publications. Ann Nutr Metab.

[5] Bakewell L, Burdge GC, Calder PC (2006). Polyunsaturated fatty acid concentrations in young men and women consuming their habitual diets. Br J Nutr.

[6] Giltay EJ, Gooren LJ, Toorians AW, Katan MB, Zock PL (2004). Docosahexaenoic acid concentrations are higher in women than in men because of estrogenic effects. Am J Clin Nutr.

[7] Giltay EJ, Duschek EJ, Katan MB, Zock PL, Neele SJ, Netelenbos JC (2004). Raloxifene and hormone replacement therapy increase arachidonic acid and docosahexaenoic acid levels in postmenopausal women. J Endocrinol.

[8] Sumino H, Ichikawa S, Murakami M, Nakamura T, Kanda T, Sakamaki T, Mizunuma H, Kurabayashi M (2003). Effects of hormone replacement therapy on circulating docosahexaenoic acid and eicosapentaenoic acid levels in postmenopausal women. Endocr J.

[9] Arterburn LM, Hall EB, Oken H (2006). Distribution, interconversion, and dose response of n-3 fatty acids in humans. Am J Clin Nutr.

[10] Brenna JT, Salem N, Sinclair AJ, Cunnane SC (2009). alpha-Linolenic acid supplementation and conversion to n-3 long-chain polyunsaturated fatty acids in humans. Prostaglandins Leukot Essent Fatty Acids.

[11] Burdge GC, Calder PC (2006). Dietary alpha-linolenic acid and health-related outcomes: a metabolic perspective. Nutr Res Rev.

[12] Finnegan YE, Minihane AM, Leigh-Firbank EC, Kew S, Meijer GW, Muggli R, Calder PC, Williams CM (2003). Plant- and marine-derived n-3 polyunsaturated fatty acids have differential effects on fasting and postprandial blood lipid concentrations and on the susceptibility of LDL to oxidative modification in moderately hyperlipidemic subjects. Am J Clin Nutr.

[13] Scientific Advisory Committee on Nutrition/Committee on Toxicity (2004). Advice on Fish Consumption: Benefits & Risks.

